# Machine learning identified EPHB2 and TOP2A as key genes linking systemic lupus erythematosus to colorectal cancer

**DOI:** 10.1097/MD.0000000000044521

**Published:** 2025-09-12

**Authors:** Guojiang Tian, Jianfei Huang

**Affiliations:** a Proctology, Shaoxing People’s Hospital, Shaoxing, Zhejiang Province, China.

**Keywords:** colorectal cancer, EPHB2, immune infiltration, machine learning, systemic lupus erythematosus, TOP2A

## Abstract

Colorectal cancer (CRC) is a leading global health burden. Systemic lupus erythematosus (SLE) is associated with a higher risk of CRC, but the molecular links between these diseases remain unclear. This study aims to identify key genes that connect SLE to CRC using machine learning approaches. We integrated genomic data from SLE and CRC patients and applied computational methods to uncover shared genetic signatures. Differential expression analysis, weighted gene co-expression network analysis (WGCNA), and machine learning techniques were used to identify hub genes. gene ontology and kyoto encyclopedia of genes and genomes enrichment analyses were performed on shared genes. Additionally, immune infiltration analysis and gene set enrichment analysis were conducted to explore the potential roles of the identified genes. Our analysis revealed 12 shared genes between SLE and CRC, with EPHB2 and TOP2A emerging as key hub genes. EPHB2 and TOP2A were significantly overexpressed in both diseases, suggesting their role in inflammatory and tumorigenic processes. EPHB2 showed excellent diagnostic performance in SLE, while high EPHB2 expression was associated with better overall survival in CRC patients. gene set enrichment analysis identified pathways associated with these hub genes, implicating them in immune response, cell cycle regulation, and DNA replication. Moreover, EPHB2 and TOP2A were found to be associated with immune infiltration in CRC. EPHB2 and TOP2A serve as bridge genes linking SLE and CRC, offering insights into their molecular interplay and the potential for developing new diagnostic markers and therapeutic targets. Future studies should validate these findings and explore the detailed molecular mechanisms.

## 1. Introduction

Colorectal cancer (CRC) is one of the most common malignancies worldwide, with a significant burden on global health systems.^[1]^ Despite advances in early detection and treatment, CRC remains a leading cause of cancer-related mortality.^[2]^ The disease arises from a complex interplay of genetic, environmental, and lifestyle factors, which contribute to the transformation of normal colonic epithelial cells into malignant tumors. Genetic alterations, such as mutations in oncogenes and tumor suppressor genes, play a crucial role in CRC development.^[3]^ Additionally, chronic inflammation has been identified as a key driver of colorectal carcinogenesis, with conditions like inflammatory bowel disease significantly increasing the risk of CRC.^[4]^ Understanding the molecular mechanisms underlying CRC progression is essential for developing more effective prevention strategies and personalized therapies. Recent advances in genomics and machine learning have provided powerful tools to identify novel biomarkers and therapeutic targets,^[5]^ offering new insights into the pathogenesis of CRC.

Systemic lupus erythematosus (SLE) is a chronic autoimmune disease characterized by systemic inflammation and multi-organ involvement.^[6]^ SLE affects multiple body systems, including the skin, joints, kidneys, heart, and lungs, and is associated with a higher risk of various cancers, including CRC.^[7]^ The exact mechanisms linking SLE to CRC are not fully understood, but chronic inflammation, immune dysregulation, and genetic predisposition are believed to play important roles.^[8,9]^ Studies have shown that patients with SLE have a 1.2- to 2.18-fold increased risk of developing CRC compared to the general population.^[7]^ This heightened risk may be attributed to the prolonged inflammatory state in SLE, which can promote genomic instability and enhance the likelihood of carcinogenic mutations.^[10,11]^ Moreover, the use of immunosuppressive drugs to manage SLE may further contribute to cancer risk by impairing the body’s natural immune surveillance against neoplastic cells.^[12,13]^ Therefore, elucidating the molecular connections between SLE and CRC is critical for identifying potential therapeutic interventions and improving patient outcomes.

**Figure 1.**
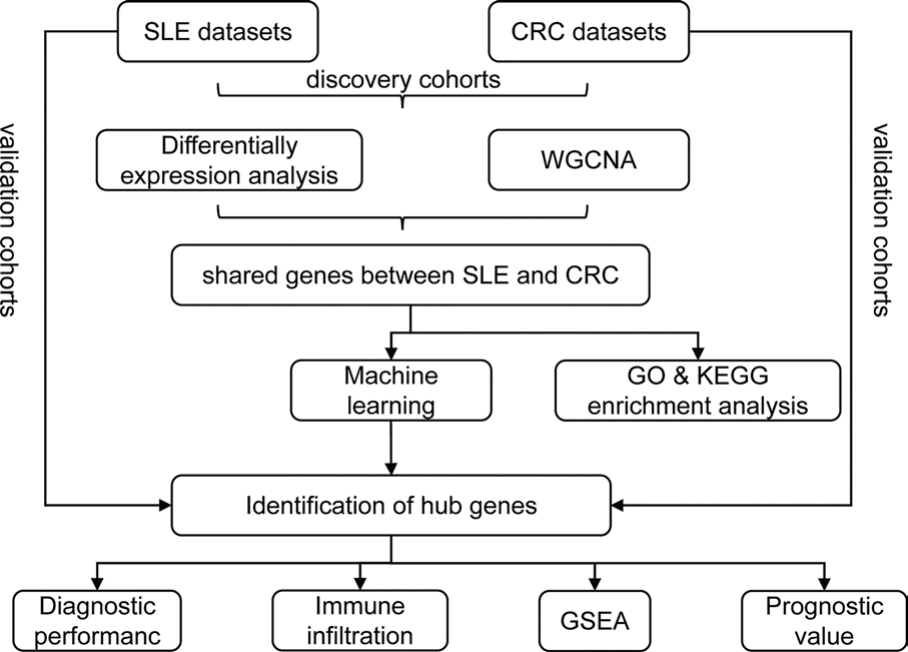
The workflow diagram of identifying shared genes between creasing the risk of (CRC) and SLE. CRC = colorectal cancer, SLE = systemic lupus erythematosus.

In this study, we aim to identify key genes that link SLE to CRC using machine learning approaches. We integrated large-scale genomic data from SLE and CRC patients and applied advanced computational methods to identify shared genetic signatures underlying the increased risk of CRC in SLE. Our analysis has highlighted 2 genes, EPHB2 and TOP2A, as potential bridging factors between these 2 diseases. EPHB2, a member of the Eph receptor family, is involved in cell-cell signaling and has been implicated in various cancers, including CRC. TOP2A, which encodes topoisomerase II alpha, is essential for DNA replication and repair and has been linked to both SLE and CRC. The discovery of these key genes lays the groundwork for in-depth studies of the molecular links between SLE and CRC. This work may ultimately enable the development of novel diagnostic markers and targeted therapies. The workflow diagram is shown in Figure 1.

## 2. Materials and methods

### 2.1. Data collection and processing

Expression matrix files of peripheral blood mononuclear cell samples from 7 SLE cohorts were downloaded from the GEO database (https://www.ncbi.nlm.nih.gov/geo/), including GSE65391, GSE121239, GSE61635, GSE4588, GSE50772, GSE99967, and GSE24706. Additionally, CRC expression matrix datasets were downloaded from the GEO database, including GSE110224, GSE41258, GSE39582, and GSE38832. Batch effects were removed using the R package “SVA.” Transcriptional and follow-up data from the TCGA-COAD cohort were downloaded from the TCGA database (https://portal.gdc.cancer.gov/), excluding cases with a follow-up time of <30 days, and the transcriptome dataset was log-transformed. All cohort information is summarized in Table 1.

**Table 1:** Summary of SLE and CRC cohorts used in this study.

Disease	GEO accession	Case	Control	Platform	Samples
SLE	GSE72326	157	20	GPL10558	peripheral blood
SLE	GSE81622	30	25	GPL10558	peripheral blood
SLE	GSE65391	924	72	GPL10558	peripheral blood
SLE	GSE121239	292	20	GPL13158	peripheral blood
SLE	GSE61635	99	30	GPL570	peripheral blood
SLE	GSE4588	15	19	GPL570	peripheral blood
SLE	GSE50772	61	20	GPL570	peripheral blood
SLE	GSE99967	42	17	GPL21970	peripheral blood
SLE	GSE24706	28	20	GPL6884	peripheral blood
CRC	GSE110224	7	7	GPL570	Colonic tissue
CRC	GSE41258	316	74	GPL96	Colonic tissue
CRC	GSE39582	566	19	GPL570	Colonic tissue
CRC	GSE38832	122		GPL570	Colonic tissue

CRC = colorectal cancer, SLE = systemic lupus erythematosus.

### 2.2. Differential expression analysis

Differentially expressed genes (DEGs) in SLE and CRC were identified using the limma package. DEGs in CRC were defined by |log2FC| >1 and adjusted *P*-value <.05, whereas in SLE they were defined by adjusted *P*-value <.05. The overall view of DEGs was presented in volcano plots, and heatmaps were used to visualize the expression patterns of DEGs. Volcano plots and heatmaps were generated using the “ggplot2” R package.

### 2.3. Weighted gene co-expression network analysis

Potential functional modules in SLE and CRC samples were identified using the R package WGCNA. Based on the weighted correlation adjacency matrix and clustering analysis, genes with similar expression patterns were assigned to co-expression modules. The topological overlap matrix was derived from the adjacency matrix, and genes were assigned to different modules based on their differences in topological overlap matrix. The cutting height was set to 0.25, and the minimum module size was set to 20. The soft-threshold power for CRC and SLE was set to 5 and 4, respectively. Additionally, gene significance and module membership were analyzed. Spearman correlation coefficients and their corresponding p-values were also calculated. Finally, relevant genes from central modules were extracted for further analysis.

### 2.4. Shared gene identification and enrichment analysis

We identified SLE- and CRC-related modules via WGCNA and extracted their most up- and down-regulated genes. The overlap between these 2 gene sets yielded the shared genes. The intersection of these genes resulted in shared genes. The clusterProfiler package was used to perform Gene Ontology (GO) and Kyoto Encyclopedia of Genes and Genomes (KEGG) enrichment analyses on the shared genes.

### 2.5. Machine learning identification of hub genes

Machine learning analysis was conducted on the shared genes in both CRC and SLE cohorts to screen for hub genes. Three machine learning techniques – least absolute shrinkage and selection operator (LASSO), support vector machine (SVM), and random forest – were used, each employing 10-fold cross-validation to ensure model stability. The R packages glmnet, e1071, caret, and random forest were utilized. We retained only those genes selected by all 3 methods (LASSO, SVM, random forest) as candidate hub genes. Finally, the genes common to both CRC and SLE analyses were defined as shared hub genes.

### 2.6. Immune infiltration analysis

The IOBR package was used to evaluate immune cell infiltration in the TCGA-COAD cohort. Using CIBERSORT algorithm, we estimated the relative abundance of 22 immune cell types and calculated Pearson correlations between these infiltration levels and hub-gene expression.

### 2.7. Gene set enrichment analysis

The cluster profiler package was used to perform gene set enrichment analysis (GSEA) to identify KEGG pathways associated with hub genes. GSEA is a category-based method that calculates the enrichment score of gene sets and discovers different functional phenotypes. Patients with SLE and CRC were divided into high and low expression groups based on the median expression levels of hub genes. The biological pathways between these 2 groups were compared using GSEA. Pathways with nominal *P*-values <.05, absolute normalized enrichment scores >1, and false discovery rate <0.25 were considered significant. We considered pathways significant if they were consistently enriched in at least 3 cohorts. Significant pathways were visualized with ggplot2.

## 3. Statistical analysis

All statistical analyses were performed using R software (version 4.3.0). Differential expression analysis was conducted using the limma package with Benjamini-Hochberg adjustment for multiple testing. Diagnostic performance of hub genes was evaluated using receiver operating characteristic (ROC) curves, with area under the curve (AUC) calculated via the pROC package. AUC values are reported with 95% confidence intervals. Kaplan–Meier survival curves were compared using log-rank tests. Immune cell infiltration correlations were assessed using Pearson correlation. In diagnostic biomarker evaluation, AUC >0.7 was deemed clinically acceptable based on established criteria (e.g., AUC 0.7–0.8: moderate accuracy; >0.8: high accuracy).

## 4. Results

### 4.1. Differentially expressed genes and modules related to CRC

**Figure 2.**
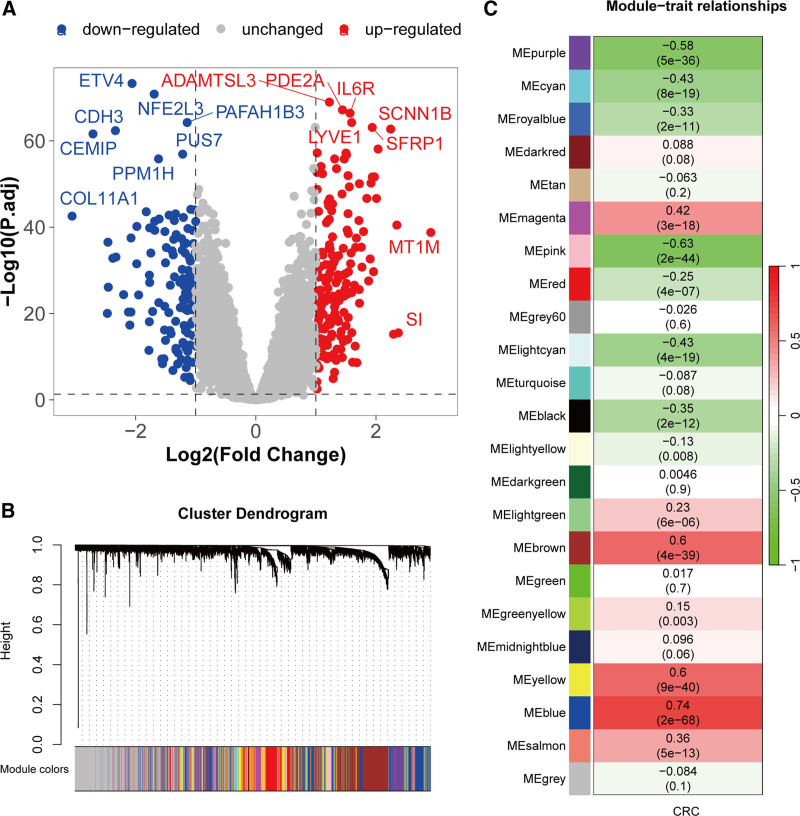
Differential expression analysis and WGCNA to identify CRC-related genes. (A) Volcano plot of differential expression analysis in the GSE41258 cohort. Genes with adjusted *P*-value <.05 (Benjamini–Hochberg correction) and |log_2_FC| >1 are highlighted (red: up-regulated; blue: downregulated). Statistical significance was tested using the limma package. (B) Dendrogram of co-expression network module clustering in CRC. (C) Module-trait relationships map. CRC = colorectal cancer, WGCNA = weighted gene co-expression network analysis.

Based on the differential expression analysis of the GSE41258 cohort, 959 genes were found to be abnormally lowly expressed, while 867 genes were abnormally highly expressed in CRC (Fig. 2A). Further WGCNA analysis of this dataset clustered all samples, and the optimal soft-power value for GSE41258 was 5 (Fig. S1, Supplemental Digital Content, https://links.lww.com/MD/P948). A total of 23 modules were identified (Fig. 2B). Subsequently, the correlations between modules and clinical traits were calculated. Modules with *R* >0.5 and *P* <.05 were selected, including purple, pink, brown, yellow, and blue, comprising 3126 genes (Fig. 2C). These findings highlight a substantial number of DEGs in CRC. WGCNA identified key modules strongly correlated with disease traits, suggesting that these modules and genes play critical roles in CRC pathogenesis by potentially regulating disease-specific pathways and networks.

### 4.2. Differentially expressed genes and modules related to SLE

**Figure 3.**
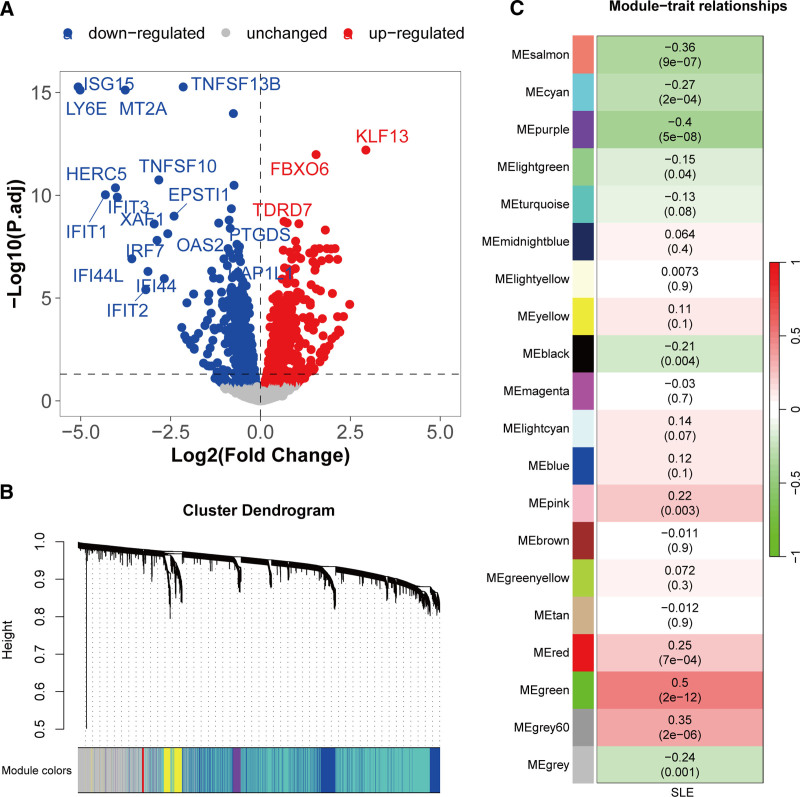
Differential expression analysis and WGCNA to identify SLE-related genes. (A) Volcano plot of differential expression analysis in the GSE72326 cohort. Genes with adjusted *P*-value <.05 (Benjamini–Hochberg correction) are highlighted (red: up-regulated; blue: downregulated). Statistical significance was tested using the limma package. (B) Dendrogram of co-expression network module clustering in SLE. (C) Module-trait relationships map. SLE = systemic lupus erythematosus, WGCNA = weighted gene co-expression network analysis.

Based on the differential expression analysis of the GSE72326 cohort, 1420 genes were found to be abnormally lowly expressed, while 1112 genes were abnormally highly expressed in SLE (Fig. 3A). Further WGCNA analysis of this dataset clustered all samples, and the optimal soft-power value for GSE41258 was 4 (Fig. S2, Supplemental Digital Content, https://links.lww.com/MD/P948). A total of 20 modules were identified (Fig. 3B). Subsequently, the correlations between modules and clinical traits were calculated. Modules with *R* >0.2 and *P* <.05 were selected, including salmon, cyan, purple, black, pink, red, green, grey60, and gray, comprising 3019 genes (Fig. 3C). The analysis reveals extensive gene dysregulation in SLE, with WGCNA modules showing moderate to strong trait correlations, indicating that these genes and modules are likely involved in immune dysregulation and inflammatory processes central to SLE development.

### 4.3. Shared genes between CRC and SLE

**Figure 4.**
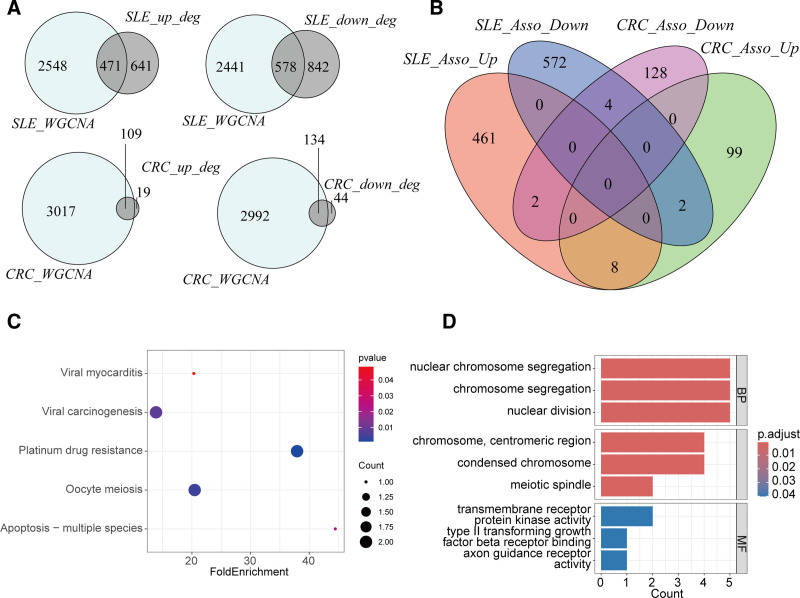
Identification of shared genes between CRC and SLE. (A) Venn diagram of SLE and CRC-related genes. (B) Venn diagram identifying shared genes between CRC and SLE. (C) KEGG enrichment analysis of shared genes. (D) GO annotation enrichment analysis of shared genes. CRC = colorectal cancer, GO = gene ontology, KEGG = Kyoto encyclopedia of genes and genomes, SLE = systemic lupus erythematosus.

To identify shared genes between CRC and SLE, we intersected the DEGs and WGCNA results from both diseases. As shown in Figure 4A, 471 highly expressed and 578 lowly expressed genes related to SLE, and 109 highly expressed and 134 lowly expressed genes related to CRC were identified. By intersecting these gene sets, we identified 8 shared highly expressed genes and 4 shared lowly expressed genes between CRC and SLE (Fig. 4B, Table 2). KEGG pathway enrichment analysis linked these genes to viral myocarditis, viral carcinogenesis, platinum drug resistance, oocyte meiosis, and apoptosis (Fig. 4C). GO enrichment analysis revealed involvement in nuclear chromosome segregation, nuclear division, transmembrane receptor protein kinase activity, type II transforming growth factor beta receptor binding, and axon guidance receptor activity (Fig. 4D). The identification of these 12 shared genes highlights molecular overlaps between CRC and SLE, with their functional roles potentially contributing to comorbidity via pathways related to cell proliferation, apoptosis, and immune responses.

**Table 2:** The shared genes between CRC and SLE.

Symbol	Gene name	Regulation
TGFBR3	Transforming growth factor beta receptor 3	Down-regulated
PID1	Phosphotyrosine interaction domain containing 1	Down-regulated
MAGEH1	MAGE family member H1	Down-regulated
SGCE	Sarcoglycan epsilon	Down-regulated
CDC20	Cell division cycle 20	Up-regulated
TOP2A	DNA topoisomerase II alpha	Up-regulated
NCAPG	Non-SMC condensin I complex subunit G	Up-regulated
IGF2BP3	Insulin like growth factor 2 mRNA binding protein 3	Up-regulated
EPHB2	EPH receptor B2	Up-regulated
ASPM	assembly factor for spindle microtubules	Up-regulated
PMAIP1	Phorbol-12-myristate-13-acetate-induced protein 1	Up-regulated
AURKA	Aurora kinase A	Up-regulated

CRC = colorectal cancer, SLE = systemic lupus erythematosus, SVM = support vector machine.

### 4.4. Hub-gene identification between CRC and SLE

**Figure 5.**
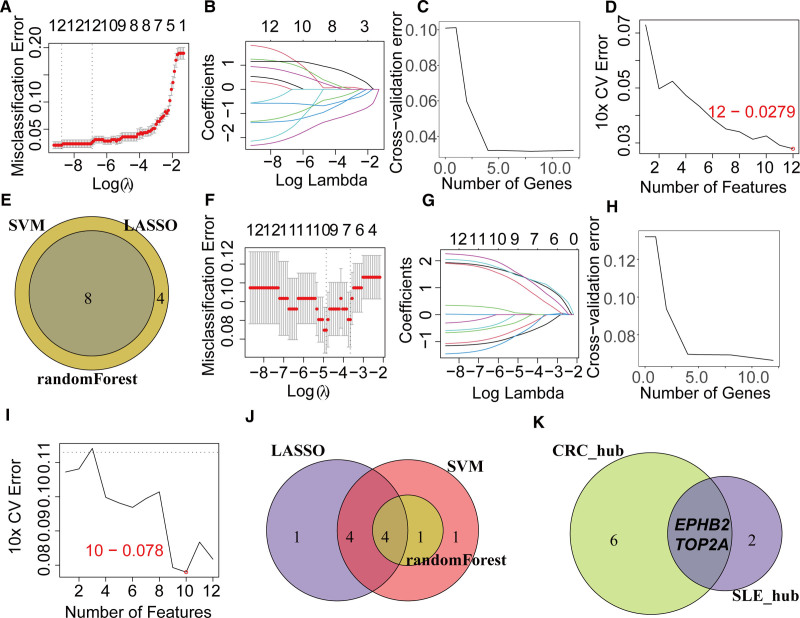
Identification of hub genes between CRC and SLE. (A) Confidence intervals at different lambda values in LASSO regression in the GSE41258 cohort. (B) Trajectory of independent variables in LASSO regression in the GSE41258 cohort. (C) Cross-validation error distribution in the random forest algorithm in the GSE41258 cohort. (D) Cross-validation error distribution in the SVM algorithm in the GSE41258 cohort. (E) Venn diagram identifying 8 hub genes in CRC. (F) Confidence intervals at different lambda values in LASSO regression in the GSE72326 cohort. (G) Trajectory of independent variables in LASSO regression in the GSE72326 cohort. (H) Cross-validation error distribution in the random forest algorithm in the GSE72326 cohort. (I) Cross-validation error distribution in the SVM algorithm in the GSE72326 cohort. (J) Venn diagram identifying 4 hub genes in SLE. (K) Venn diagram identifying 2 shared hub genes between CRC and SLE. CRC = colorectal cancer, LASSO = least absolute shrinkage and selection operator, SLE = systemic lupus erythematosus, SVM = support vector machine.

To further screen for hub genes between CRC and SLE, we utilized machine learning algorithms. The results showed that LASSO, randomForest, and SVM identified 12, 8, and 12 hub genes in CRC, respectively (Fig. 5A–D, Table 3). Taking the intersection, we obtained 8 hub genes in CRC (Fig. 5E). In SLE, the 3 algorithms identified 9, 5, and 10 hub genes, respectively (Fig. 5F–I, Table 3). Taking the intersection, we obtained 4 hub genes in SLE (Fig. 5J). Intersecting hub genes identified in CRC and SLE, we found EPHB2 and TOP2A as shared key hubs (Fig. 5K). These results imply that EPHB2 and TOP2A play central roles in the regulatory networks of both diseases and may drive disease progression via interconnected biological pathways.

**Table 3:** Hub genes identified by LASSO, random forest and SVM.

Disease	Machine learning		
	LASSO	Random forest	SVM
CRC	TOP2A, AURKA, EPHB2, PID1, CDC20, TGFBR3, PMAIP1, ASPM, MAGEH1, SGCE, NCAPG, IGF2BP3	TGFBR3, SGCE, PMAIP1, PID1, AURKA, EPHB2, TOP2A, MAGEH1	TOP2A, AURKA, EPHB2, PID1, CDC20, TGFBR3, PMAIP1, ASPM, MAGEH1, SGCE, NCAPG, IGF2BP3
SLE	TGFBR3, PID1, MAGEH1, EPHB2, TOP2A, AURKA, SGCE, NCAPG, IGF2BP3	EPHB2, NCAPG, CDC20, IGF2BP3, TOP2A	NCAPG, IGF2BP3, TOP2A, EPHB2, SGCE, PID1, TGFBR3, PMAIP1, MAGEH1, CDC20

CRC = colorectal cancer, LASSO = least absolute shrinkage and selection operator, SLE = systemic lupus erythematosus, SVM = support vector machine.

### 4.5. Expression validation of hub genes

**Figure 6.**
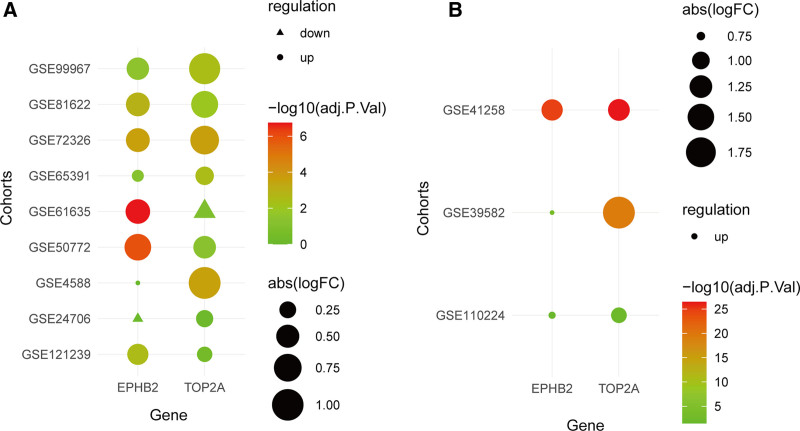
Expression validation of EPHB2 and TOP2A in (A) SLE cohorts and (B) colorectal cancer cohorts. SLE = systemic lupus erythematosus.

To validate the expression of EPHB2 and TOP2A, we performed differential expression analysis on SLE and CRC cohorts, reporting logFC and adjusted p-values. Results showed that EPHB2 and TOP2A were significantly up-regulated in most SLE cohorts (Fig. 6A) and also highly expressed in CRC (Fig. 6B). These findings confirm their abnormal overexpression in both diseases, supporting their potential as biomarkers and implicating them in proliferative and oncogenic processes.

### 4.6. Diagnostic performance of hub genes in SLE

**Figure 7.**
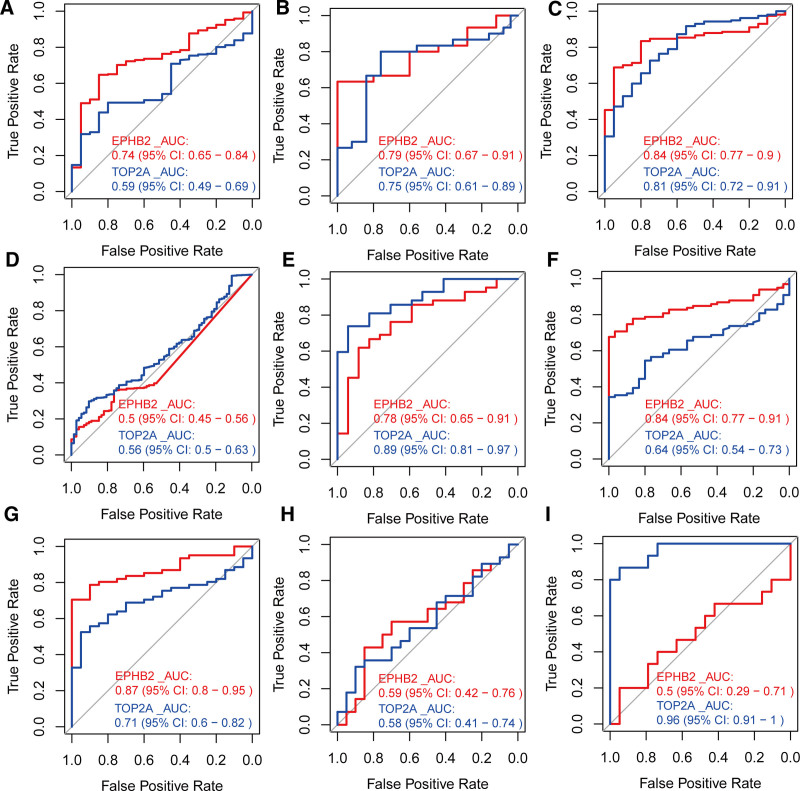
Diagnostic performance of EPHB2 and TOP2A in SLE cohorts. ROC curves with AUC values and 95% CIs for: (A) GSE121239, (B) GSE81622, (C) GSE72326, (D) GSE65391, (E) GSE99967, (F) GSE61635, (G) GSE50772, (H) GSE24706, (I) GSE4588. AUC = area under the curve, CIs = confidence intervals, ROC = receiver operating characteristic, SLE = systemic lupus erythematosus.

We evaluated the diagnostic value of EPHB2 and TOP2A in SLE using ROC curve analysis (Fig. 7A–I). The results showed that EPHB2 exhibited excellent diagnostic accuracy in 6 cohorts, including GSE121239 (AUC = 0.74, 95%CI: 0.65–0.84), GSE81622 (AUC = 0.79, 95%CI: 0.67–0.91), GSE72326 (AUC = 0.84, 95%CI: 0.77–0.9), GSE99967 (AUC = 0.78, 95%CI: 0.65–0.91), GSE61635 (AUC = 0.84, 95%CI: 0.77–0.91), and GSE50772 (AUC = 087, 95%CI: 0.8–0.95), with an AUC value >0.7, indicating its strong diagnostic performance. Additionally, TOP2A demonstrated excellent diagnostic accuracy in GSE81622 (AUC = 0.75, 95%CI: 0.61–0.89), GSE72326 (AUC = 0.81, 95%CI: 0.72–0.91), GSE99967 (AUC = 0.89, 95%CI: 0.81–0.97), GSE50772 (AUC = 0.71, 95%CI: 0.6–0.82), and GSE4588 (AUC = 0.96, 95%CI: 0.91–1), with an AUC value >0.7 in these cohorts. The high AUC values of EPHB2 and TOP2A in SLE cohorts demonstrate their strong diagnostic potential. These genes may assist early detection and disease monitoring by indicating underlying molecular changes in immune and cellular dysregulation.

### 4.7. Prognostic value of hub genes in CRC

**Figure 8.**
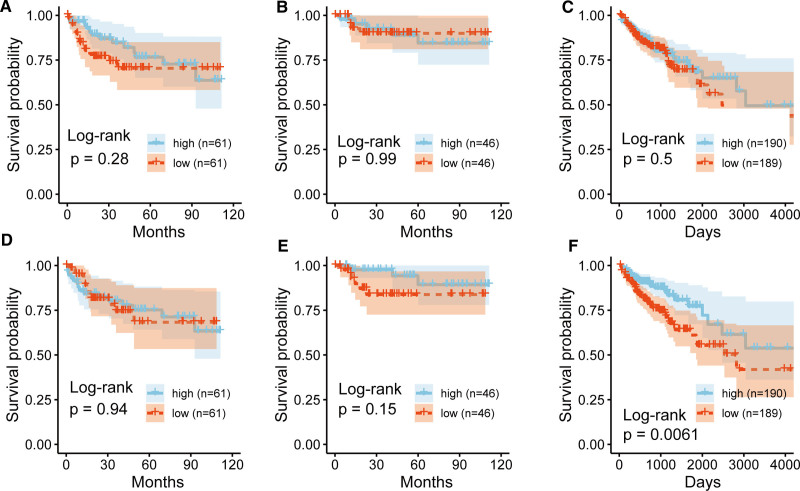
Kaplan–Meier survival curves comparing high/low expression groups. *P*-values calculated by log-rank test; data points represent observed events. Kaplan–Meier curves (A) disease-specific survival (DSS, high/low n = 61/61), (B) disease-free survival (DFS; high/low n = 46/46), and (C) overall survival (OS; high/low n = 190/189) in the GSE38832 cohort for TOP2A. KM survival curves for (D) DSS (high/low n = 61/61), (E) DFS (high/low n = 46/46), and (F) OS (high/low n = 190/189) in the TCGA-COAD cohort for EPHB2. DFS = disease-free survival, DSS = disease-specific survival, OS = overall survival.

We assessed the prognostic significance of the hub genes in CRC using the GSE38832 and TCGA-COAD cohorts. Kaplan–Meier survival analysis revealed no significant differences in disease-specific survival, disease-free survival, or overall survival (OS) between the high and low expression groups of TOP2A in the GSE38832 cohort (Fig. 8A–C). Similarly, there were no significant differences in disease-specific survival and disease-free survival between the high and low expression groups of EPHB2 in the GSE38832 cohort (Fig. 8D and E). However, patients with high EPHB2 expression had a significantly better OS compared to those with low expression in the TCGA-COAD cohort (Fig. 8F). TOP2A expression showed no significant prognostic impact. However, high EPHB2 expression was associated with improved overall survival in CRC, suggesting that EPHB2 may affect long-term outcomes via protective roles in tumor biology or therapeutic response.

### 4.8. Pathway analysis related to hub genes

**Figure 9.**
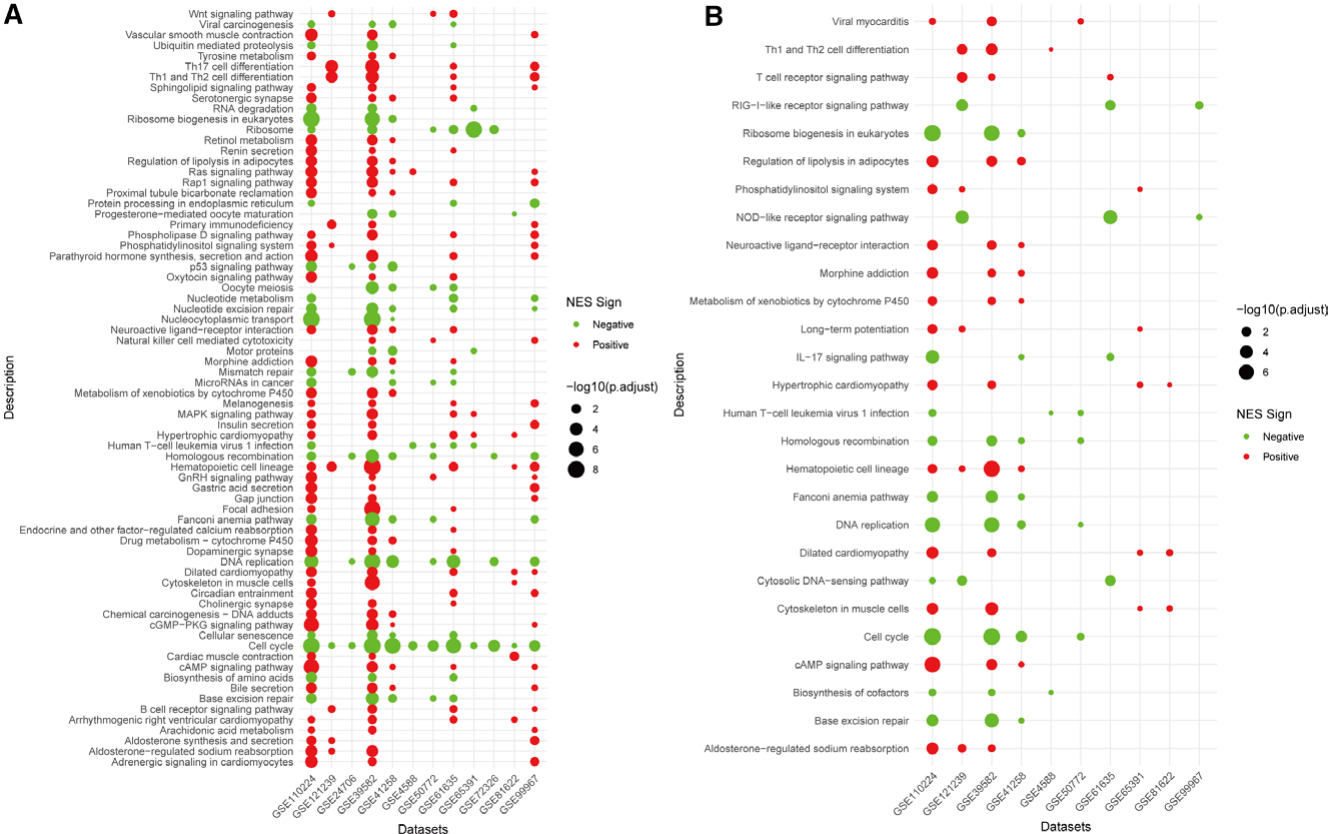
Pathway analysis related to hub genes. (A) Gene set enrichment analysis for pathways associated with TOP2A. (B) Gene set enrichment analysis for pathways associated with EPHB2.

To explore the underlying mechanisms by which EPHB2 and TOP2A link SLE and CRC, we performed GSEA based on the expression levels of these hub genes. We identified 72 pathways associated with TOP2A, of which 23 were significantly downregulated in the high expression group (e.g., nucleotide metabolism, DNA replication, cell cycle), and 49 were significantly up-regulated (e.g., T-cell differentiation-related pathways, MAPK, Ras, Rap1 signaling pathways) (Fig. 9A). For EPHB2, we identified 27 pathways. In the high expression group, 12 pathways were significantly inhibited (e.g., RIG-I receptor signaling pathway, NOD-like receptor signaling pathway, IL-17 signaling pathway, DNA replication), while 15 pathways were significantly activated, including T-cell differentiation pathways, T-cell receptor signaling pathway, phosphatidylinositol signaling system (Fig. 9B). GSEA indicates that TOP2A and EPHB2 regulate pathways involved in cell cycle control and immune activation. This dual role may explain how their dysregulation links autoimmune mechanisms in SLE with tumor development in CRC.

### 4.9. Correlation of hub genes with immune cell infiltration in CRC

**Figure 10.**
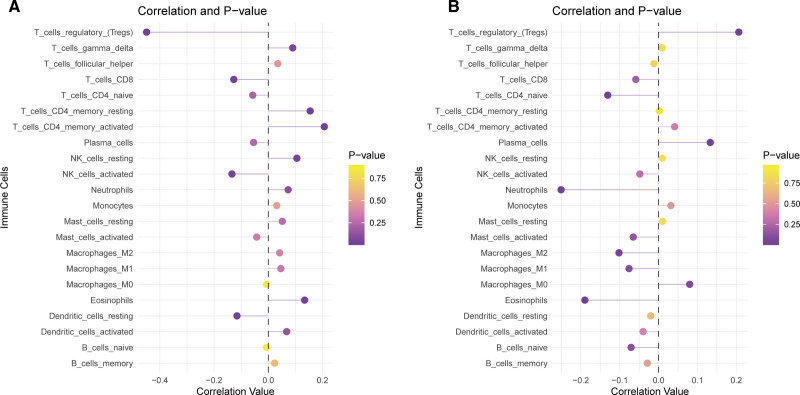
Correlation analysis of hub genes with immune cell infiltration in CRC. (A) Correlation between TOP2A expression and immune cell infiltration. (B) Correlation between EPHB2 expression and immune cell infiltration. CRC = colorectal cancer.

We further evaluated the correlation between the expression of hub genes and immune cell infiltration in the TCGA-COAD cohort. The results showed that TOP2A expression was significantly negatively correlated with regulatory T cells (Tregs), CD8 + T cells, activated natural killer cells, and resting dendritic cells, while it was positively correlated with resting and activated CD4 + T cells, eosinophils, and other immune cell types (Fig. 10A). EPHB2 expression was positively correlated with Tregs and plasma cells but negatively correlated with naive CD4 + T cells, neutrophils, eosinophils, and other cell types (Fig. 10B). These findings indicate that the hub genes are associated with specific patterns of immune cell infiltration in CRC. The differing correlations of TOP2A and EPHB2 with immune infiltrates underscore their distinct roles in shaping the tumor microenvironment. These effects may explain immune evasion or activation in CRC and connect to SLE’s autoimmune features via shared immunomodulation.

## 5. Discussion

Machine learning has become a powerful tool for discovering biomarkers in SLE comorbidity research. It has significantly advanced our understanding of shared mechanisms and revealed new therapeutic strategies.^[14,15]^ Building upon this foundation, our study utilized machine learning algorithms to elucidate shared biomarkers between SLE and colorectal cancer (CRC). This approach identified 12 shared genes, with EPHB2 and TOP2A emerging as robust shared hub genes. Critically, these hub genes demonstrated consistently elevated diagnostic performance across multiple independent cohorts, achieving AUC values ranging from 0.7 to 0.9. This indicates moderate to good diagnostic accuracy for distinguishing SLE patients from controls. It is well-established that an AUC > 0.7 represents the widely accepted minimum threshold for clinical utility of a biomarker.^[16,17]^ Biomarkers within the AUC 0.7 to 0.8 range can support clinical decision-making for intermediate-risk patient stratification, serve as preliminary predictors of treatment response, and function effectively as adjunctive diagnostic tools. Furthermore, biomarkers achieving AUC values exceeding 0.8 possess sufficient discriminatory power to be considered potential stand-alone diagnostic tools. These results shed light on the molecular links between SLE and CRC. They also lay a theoretical foundation for creating new diagnostic biomarkers and therapeutic targets.

KEGG and GO enrichment analyses of the shared genes revealed significant involvement in multiple biological processes and molecular functions, including viral myocarditis, viral carcinogenesis, platinum drug resistance, oocyte meiosis, apoptosis, nuclear chromosome segregation, nuclear division, transmembrane receptor protein kinase activity, type II transforming growth factor beta receptor binding, and axon guidance receptor activity. These findings suggest that SLE and CRC may share common pathological mechanisms, particularly in immune response and cell cycle regulation.

EPHB2, a member of the Eph receptor family, plays a crucial role in cell-cell signaling and has been implicated in various cancers, including CRC.^[18]^ TOP2A, encoding topoisomerase II alpha, is essential for DNA replication and repair and has been linked to both SLE and CRC.^[19,20]^ Our study confirmed that both EPHB2 and TOP2A are significantly overexpressed in SLE and CRC patients, suggesting their potential role as bridges in the inflammatory and tumorigenic processes. EPHB2, a receptor tyrosine kinase in the Eph/ephrin family, facilitates bidirectional signaling that regulates cell migration and adhesion. In CRC, it promotes tumor invasiveness by controlling the crypt-villous expression gradient and epithelial homeostasis.^[21,22]^ In SLE-like autoimmune settings, EPHB2 drives immune cell activation and fibrosis, as evidenced in systemic sclerosis where it enhances extracellular matrix deposition and inflammation.^[23]^ This implies that SLE’s chronic inflammatory state could upregulate EPHB2, bridging to CRC by enabling immune-mediated epithelial disruption and metastasis. Similarly, TOP2A resolves DNA supercoiling to prevent genomic instability; its overexpression in CRC supports proliferation and chemoresistance via pathway modulation.^[24–26]^ In SLE, defective DNA repair exacerbates damage, where TOP2A’s catenation monitoring may falter under oxidative stress, potentially priming colorectal cells for oncogenic mutations.^[27,28]^ Thus, these genes mechanistically link SLE’s immune and DNA repair defects to CRC’s proliferative and invasive traits, highlighting shared inflammatory-genomic pathways.

EPHB2 showed excellent diagnostic performance in SLE, while TOP2A did not show a significant association with CRC prognosis. However, high EPHB2 expression was associated with better overall survival in CRC patients, indicating its potential as a biomarker for both SLE and CRC. In cancer research, EPHB2 has been linked to DNA repair, stem cell characteristics, and epigenetic regulation, including DNA methylation and RNA modifications.^[29–31]^ Moreover, EPHB2 is involved in immune responses and cancer pathways, with high expression associated with epithelial-mesenchymal transition activation in various cancers.^[32,33]^ In SLE, the presence of anti-EPHB2 autoantibodies in 56% of serum samples suggests that EPHB2 is highly recognized in SLE patients, with a specificity of 95% and sensitivity of 35%.^[34]^ This indicates its potential as a diagnostic tool for SLE.

Further analysis revealed that EPHB2 is associated with RIG-I receptor, NOD-like receptor, and IL-17 signaling pathways. The RIG-I receptor pathway is critical for recognizing viral RNA and triggering innate immune responses,^[35]^ while NOD-like receptors recognize bacterial pathogen-associated molecular patterns and activate inflammatory responses.^[36]^ The IL-17 signaling pathway is a pro-inflammatory pathway involved in various autoimmune diseases, including SLE.^[37]^ In CRC, abnormal immune responses in the tumor microenvironment may be influenced by EPHB2’s involvement in these immune-related pathways, potentially modulating the tumor immune microenvironment. Moreover, EPHB2 is associated with T-cell differentiation pathways, the T-cell receptor (TCR) signaling pathway, and DNA replication. The T-cell differentiation and TCR signaling pathways are critical for the normal functioning of the immune system. In systemic lupus erythematosus (SLE), T-cell function is aberrant, characterized by the uncontrolled activation and proliferation of autoreactive T cells.^[38]^ In colorectal cancer (CRC), tumor cells can evade immune surveillance by modulating T-cell function. The involvement of EPHB2 in these pathways suggests its potential role in coordinating the dysregulated T-cell functions observed in both SLE and CRC.

TOP2A is associated with nucleotide metabolism, DNA replication, and cell cycle-related pathways. In normal cellular function, nucleotide metabolism provides the building blocks for DNA replication, a key process in the cell cycle. In CRC, dysregulation of the cell cycle is a hallmark of tumor cell proliferation.^[39]^ The involvement of TOP2A in these pathways may imply its critical role in the proliferation of CRC cells. While cell proliferation is not a primary pathological feature of SLE, autoimmune reactions can influence intracellular metabolism and cell cycle progression. Aberrations in TOP2A may have a potential link to the pathogenesis of SLE, suggesting that it could contribute to disease mechanisms by affecting cellular processes. T-cell differentiation pathways are crucial for the development and function of T cells. Signaling pathways such as MAPK, Ras, and Rap1 play extensive roles in various cellular functions, including proliferation, differentiation, survival, and immune regulation.^[40–43]^ In CRC, aberrant activation of these signaling pathways can promote tumor growth and immune evasion.^[44]^ In SLE, they may be involved in the activation of autoreactive T cells and the maintenance of autoimmune responses.^[45]^ The association of TOP2A with these pathways could represent a significant factor linking the immune and cellular dysfunctions observed in both CRC and SLE.

Our study also demonstrated that the expression levels of EPHB2 and TOP2A are closely correlated with specific types of immune cell infiltration in CRC. Previous research has shown that the forward signaling of EPHB2, through specific binding to ephrin-B1/B2, can promote the activation of monocytes and the migration of T cells.^[46]^ Additionally, the transdifferentiation of monocytes into macrophages is associated with increased EPHB2 expression, and monocytes exposed to immobilized ephrinB2 exhibit enhanced receptor phosphorylation, leading to up-regulated expression of pro-inflammatory chemokines such as MCP-1/CCL2 and IL-8.^[47]^ A study by Yu et al revealed that EPHB2 is involved in the activation of human naive B cells via the Notch1 and Src-p65 signaling pathways and is regulated by miR-185.^[48]^ These findings suggest that EPHB2 may play a significant role in tumor immunity, warranting further exploration in future research.

Given the identification of EPHB2 and TOP2A as hub genes linking SLE and CRC, pharmacological targeting offers promising therapeutic avenues. For EPHB2, small-molecule inhibitors like STA-013,^[49]^ dasatinib^[50]^ and GLPG1790 (pan-Eph antagonist reducing cancer stem cells)^[51]^ are available, demonstrating antitumor effects in CRC and potential to modulate autoimmune inflammation. For TOP2A, poisons such as etoposide and doxorubicin stabilize DNA cleavage complexes for antiproliferative activity,^[52,53]^ while catalytic inhibitors like ICRF193^[54]^ and TSC24^[55]^ block ATPase function with less genotoxicity, suitable for SLE’s DNA repair vulnerabilities. Importantly, these findings highlight the potential for repurposing existing drugs to address the SLE-CRC comorbidity, offering a novel strategy to improve clinical outcomes in patients with both conditions.

Despite the insights provided by this study, some limitations should be acknowledged. First, the study primarily relied on publicly available datasets, lacking independent cohort validation. Second, due to limited sample sizes, some conclusions may require confirmation through larger-scale studies. Third, while we identified EPHB2 and TOP2A as key hub genes via machine learning, these findings require experimental validation through Western blotting or qRT-PCR to confirm protein-level expression and functional relevance. Fourth, although we removed batch effects using the R package SVA, residual technical variations may persist due to the multi-cohort nature of our analysis, potentially influencing gene expression patterns. Finally, while we identified 2 key genes, the specific molecular mechanisms by which they promote the transition from SLE to CRC remain to be fully elucidated.

## 6. Conclusion

In conclusion, our study highlights the importance of EPHB2 and TOP2A as bridge genes linking SLE and CRC. These findings provide valuable insights into the molecular mechanisms underlying the relationship between these 2 diseases and offer potential targets for developing new diagnostic markers and therapeutic strategies. Future research should focus on validating these findings in independent cohorts and exploring the detailed molecular mechanisms involved.

## Author contributions

**Conceptualization:** Guojiang Tian, Jianfei Huang.

**Data curation:** Guojiang Tian, Jianfei Huang.

**Formal analysis:** Guojiang Tian, Jianfei Huang.

**Funding acquisition:** Guojiang Tian, Jianfei Huang.

**Investigation:** Guojiang Tian, Jianfei Huang.

**Methodology:** Guojiang Tian, Jianfei Huang.

**Project administration:** Guojiang Tian, Jianfei Huang.

**Resources:** Guojiang Tian, Jianfei Huang.

**Software:** Guojiang Tian, Jianfei Huang.

**Supervision:** Guojiang Tian, Jianfei Huang.

**Validation:** Guojiang Tian, Jianfei Huang.

**Visualization:** Guojiang Tian, Jianfei Huang.

**Writing – original draft:** Guojiang Tian, Jianfei Huang.

**Writing – review & editing:** Guojiang Tian, Jianfei Huang.

## Supplementary Material



## References

[1] Svec J Onhajzer J Korinek V. Origin, development and therapy of colorectal cancer from the perspective of a biologist and an oncologist. Crit Rev Oncol Hematol. 2024;204:104544. 39490796 10.1016/j.critrevonc.2024.104544

[2] Saoudi González N Ros J Baraibar I. Cetuximab as a key partner in personalized targeted therapy for metastatic colorectal cancer. Cancers (Basel). 2024;16:412. 38254903 10.3390/cancers16020412 PMC10814823

[3] Pappas L Quintanilha JCF Huang RSP Parikh AR. Genomic alterations associated with early-stage disease and early recurrence in patients with colorectal cancer. Oncologist. 2024;30:oyae269. 10.1093/oncolo/oyae269 PMC11883158 39531357

[4] Li AJ Jiang HY Jia YH. Statin exposure and risk of colorectal cancer in patients with inflammatory bowel disease: a systematic review and meta-analysis. Front Med (Lausanne). 2024;11:1507739. 39650188 10.3389/fmed.2024.1507739 PMC11624505

[5] Vaziri-Moghadam A Foroughmand-Araabi MH. Integrating machine learning and bioinformatics approaches for identifying novel diagnostic gene biomarkers in colorectal cancer. Sci Rep. 2024;14:24786. 39433800 10.1038/s41598-024-75438-6 PMC11494190

[6] Liu Y Yang X. A review on the novel biomarkers of systemic lupus erythematosus discovered via metabolomic profiling. Front Immunol. 2024;15:1443440. 39569194 10.3389/fimmu.2024.1443440 PMC11576423

[7] Zhang M Wang Y Wang Y Bai Y Gu D. Association between systemic lupus erythematosus and cancer morbidity and mortality: findings from cohort studies. Front Oncol. 2022;12:860794. 35600353 10.3389/fonc.2022.860794 PMC9115099

[8] Mielcarska S Kula A Dawidowicz M. Assessment of the RANTES level correlation and selected inflammatory and pro-angiogenic molecules evaluation of their influence on CRC clinical features: a preliminary observational study. Medicina (Kaunas). 2022;58:203. 35208526 10.3390/medicina58020203 PMC8880690

[9] Ohmes J Comdühr S Akbarzadeh R Riemekasten G Humrich JY. Dysregulation and chronicity of pathogenic T cell responses in the pre-diseased stage of lupus. Front Immunol. 2022;13:1007078. 36389689 10.3389/fimmu.2022.1007078 PMC9650673

[10] Vinuesa CG Shen N Ware T. Genetics of SLE: mechanistic insights from monogenic disease and disease-associated variants. Nat Rev Nephrol. 2023;19:558–72. 37438615 10.1038/s41581-023-00732-x

[11] Xiang M Wang Y Gao Z. Exploring causal correlations between inflammatory cytokines and systemic lupus erythematosus: a Mendelian randomization. Front Immunol. 2022;13:985729. 36741410 10.3389/fimmu.2022.985729 PMC9893779

[12] Kempen JH Newcomb CW Washington TL; Systemic Immunosuppressive Therapy for Eye Diseases Cohort Study Research Group. Use of immunosuppression and the risk of subsequent overall or cancer mortality. Ophthalmology. 2023;130:1258–68. 37499954 10.1016/j.ophtha.2023.07.023 PMC10811288

[13] Kosałka-Węgiel J Pacholczak-Madej R Dziedzic R. Malignancy in systemic lupus erythematosus: relation to disease characteristics in 92 patients - a single center retrospective study. Rheumatol Int. 2024;44:1701–13. 38850326 10.1007/s00296-024-05623-3 PMC11343918

[14] Wang K Wang S Ding Y Kou Z Jiang B Hou S. Exploring the molecular mechanisms and shared gene signatures between systemic lupus erythematosus and bladder urothelial carcinoma. Int J Gen Med. 2024;17:705–23. 38435117 10.2147/IJGM.S448720 PMC10909332

[15] Wang Y Huang Z Xiao Y Wan W Yang X. The shared biomarkers and pathways of systemic lupus erythematosus and metabolic syndrome analyzed by bioinformatics combining machine learning algorithm and single-cell sequencing analysis. Front Immunol. 2022;13:1015882. 36341378 10.3389/fimmu.2022.1015882 PMC9627509

[16] Swets JA. Measuring the accuracy of diagnostic systems. Science. 1988;240:1285–93. 3287615 10.1126/science.3287615

[17] Pepe MS Janes H Longton G Leisenring W Newcomb P. Limitations of the odds ratio in gauging the performance of a diagnostic, prognostic, or screening marker. Am J Epidemiol. 2004;159:882–90. 15105181 10.1093/aje/kwh101

[18] Xu S Zheng Y Ye M. Comprehensive pan-cancer analysis reveals EPHB2 is a novel predictive biomarker for prognosis and immunotherapy response. BMC Cancer. 2024;24:1064. 39198775 10.1186/s12885-024-12843-0 PMC11351591

[19] Sønderstrup IM Nygård SB Poulsen TS. Topoisomerase-1 and -2A gene copy numbers are elevated in mismatch repair-proficient colorectal cancers. Mol Oncol. 2015;9:1207–17. 25777966 10.1016/j.molonc.2015.02.009 PMC5528751

[20] Xiao L Xiao W Lin S. Potential biomarkers for active renal involvement in systemic lupus erythematosus patients. Front Med (Lausanne). 2022;9:995103. 36530895 10.3389/fmed.2022.995103 PMC9754094

[21] Papadakos SP Dedes N Pergaris A Gazouli M Theocharis S. The EPH/Ephrin system in colorectal cancer. Int J Mol Sci. 2022;23:3620. 35269901 10.3390/ijms23052761 PMC8910949

[22] Pasquale EB. Eph receptors and ephrins in cancer: bidirectional signalling and beyond. Nat Rev Cancer. 2010;10:165–80. 20179713 10.1038/nrc2806 PMC2921274

[23] Darling TK Lamb TJ. Emerging roles for Eph receptors and ephrin ligands in immunity. Front Immunol. 2019;10:1473. 31333644 10.3389/fimmu.2019.01473 PMC6620610

[24] Zhou T Niu Y Li Y. Advances in research on malignant tumors and targeted agents for TOP2A (Review). Mol Med Rep. 2025;31:50. 39670307 10.3892/mmr.2024.13415 PMC11653171

[25] Zhu C Zhang L Zhao S. UPF1 promotes chemoresistance to oxaliplatin through regulation of TOP2A activity and maintenance of stemness in colorectal cancer. Cell Death Dis. 2021;12:519. 34021129 10.1038/s41419-021-03798-2 PMC8140095

[26] Pommier Y Nussenzweig A Takeda S Austin C. Human topoisomerases and their roles in genome stability and organization. Nat Rev Mol Cell Biol. 2022;23:407–27. 35228717 10.1038/s41580-022-00452-3 PMC8883456

[27] Smith PJ McKeown SR Patterson LH. Targeting DNA topoisomerase IIα (TOP2A) in the hypoxic tumour microenvironment using unidirectional hypoxia-activated prodrugs (uHAPs). IUBMB Life. 2023;75:40–54. 35499745 10.1002/iub.2619 PMC10084299

[28] Fu H Tan W Chen Z. TOP2A deficit-induced abnormal decidualization leads to recurrent implantation failure via the NF-κB signaling pathway. Reprod Biol Endocrinol. 2022;20:142. 36138481 10.1186/s12958-022-01013-1 PMC9494868

[29] Carter J Hulse M Sivakumar M. PRMT5 inhibitors regulate DNA damage repair pathways in cancer cells and improve response to PARP inhibition and chemotherapies. Cancer Res Commun. 2023;3:2233–43. 37861290 10.1158/2767-9764.CRC-23-0070 PMC10627093

[30] Colletti E El Shabrawy D Soland M. EphB2 isolates a human marrow stromal cell subpopulation with enhanced ability to contribute to the resident intestinal cellular pool. FASEB J. 2013;27:2111–21. 23413357 10.1096/fj.12-205054 PMC3659358

[31] Zhang X. EphB2: a signature of colorectal cancer stem cells to predict relapse. Protein Cell. 2011;2:347–8. 21667331 10.1007/s13238-011-1058-6 PMC4875343

[32] Janes PW Vail ME Ernst M Scott AM. Eph receptors in the immunosuppressive tumor microenvironment. Cancer Res. 2021;81:801–5. 33177063 10.1158/0008-5472.CAN-20-3047

[33] Liu W Yu C Li J Fang J. The Roles of EphB2 in Cancer. Front Cell Dev Biol. 2022;10:788587. 35223830 10.3389/fcell.2022.788587 PMC8866850

[34] Azzouz DF Martin GV Arnoux F. Anti-Ephrin Type-B Receptor 2 (EphB2) and anti-three prime histone mRNA EXonuclease 1 (THEX1) autoantibodies in scleroderma and lupus. PLoS One. 2016;11:e0160283. 27617966 10.1371/journal.pone.0160283 PMC5019431

[35] Kato H Takeuchi O Sato S. Differential roles of MDA5 and RIG-I helicases in the recognition of RNA viruses. Nature. 2006;441:101–5. 16625202 10.1038/nature04734

[36] Franchi L Warner N Viani K Nuñez G. Function of Nod-like receptors in microbial recognition and host defense. Immunol Rev. 2009;227:106–28. 19120480 10.1111/j.1600-065X.2008.00734.x PMC2679989

[37] Iwakura Y Ishigame H Saijo S Nakae S. Functional specialization of interleukin-17 family members. Immunity. 2011;34:149–62. 21349428 10.1016/j.immuni.2011.02.012

[38] Tsokos GC. Systemic lupus erythematosus. N Engl J Med. 2011;365:2110–21. 22129255 10.1056/NEJMra1100359

[39] Hanahan D Weinberg RA. Hallmarks of cancer: the next generation. Cell. 2011;144:646–74. 21376230 10.1016/j.cell.2011.02.013

[40] Bradshaw T Simmons C Ott RK Armstrong AR. Ras/MAPK signaling mediates adipose tissue control of ovarian germline survival and ovulation in Drosophila melanogaster. Dev Biol. 2024;510:17–28. 38423203 10.1016/j.ydbio.2024.02.009

[41] Kyriakis JM Avruch J. Mammalian mitogen-activated protein kinase signal transduction pathways activated by stress and inflammation. Physiol Rev. 2001;81:807–69. 11274345 10.1152/physrev.2001.81.2.807

[42] Li X Qian H Ye H. DEHP induces apoptosis and autophagy of the thyroid via Rap1 signaling pathway: in vivo and in vitro study. Food Chem Toxicol. 2024;187:114609. 38522500 10.1016/j.fct.2024.114609

[43] Tang X Xu S Yang Z. EspP2 regulates the adhesion of glaesserella parasuis via Rap1 signaling pathway. Int J Mol Sci. 2024;25:4570. 38674155 10.3390/ijms25084570 PMC11050538

[44] Wu T Yu Y Tu X. Tubeimoside-I, an inhibitor of HSPD1, enhances cytotoxicity of oxaliplatin by activating ER stress and MAPK signaling pathways in colorectal cancer. J Ethnopharmacol. 2025;336:118754. 39208999 10.1016/j.jep.2024.118754

[45] Chen Y Tao T Liang Z. Prednisone combined with Dihydroartemisinin attenuates systemic lupus erythematosus by regulating M1/M2 balance through the MAPK signaling pathway. Mol Immunol. 2024;170:144–55. 38669759 10.1016/j.molimm.2024.04.011

[46] Guo S Wang Y Duan Q. Activation of EphrinB2/EphB2 signaling in the spine cord alters glia-neuron interactions in mice with visceral hyperalgesia following maternal separation. Front Pharmacol. 2024;15:1463339. 39290870 10.3389/fphar.2024.1463339 PMC11405339

[47] McClelland AC Sheffler-Collins SI Kayser MS Dalva MB. Ephrin-B1 and ephrin-B2 mediate EphB-dependent presynaptic development via syntenin-1. Proc Natl Acad Sci U S A. 2009;106:20487–92. 19915143 10.1073/pnas.0811862106 PMC2787153

[48] Yu M Liang W Wen S. EphB2 contributes to human naive B-cell activation and is regulated by miR-185. FASEB J. 2014;28:3609–17. 24803541 10.1096/fj.13-247759

[49] TareqSEwidaHAZoubiS. Discovery of pan-EphB tyrosine kinase inhibitor for metabolic syndrome sparing EphB3 signaling in mice. Pharmacol Res. 2025;219:107900.40769385 10.1016/j.phrs.2025.107900

[50] Ren J Amoozgar Z Uccello TP. Targeting EPHB2/ABL1 restores antitumor immunity in preclinical models of ependymoma. Proc Natl Acad Sci U S A. 2025;122:e2319474122. 39841145 10.1073/pnas.2319474122 PMC11789170

[51] Megiorni F Gravina GL Camero S. Pharmacological targeting of the ephrin receptor kinase signalling by GLPG1790 in vitro and in vivo reverts oncophenotype, induces myogenic differentiation and radiosensitizes embryonal rhabdomyosarcoma cells. J Hematol Oncol. 2017;10:161. 28985758 10.1186/s13045-017-0530-z PMC6389084

[52] Wang Z Zhu Q Li X. TOP2A inhibition reverses drug resistance of hepatocellular carcinoma to regorafenib. Am J Cancer Res. 2022;12:4343–60. 36225636 PMC9548008

[53] Takihira S Yamada D Osone T. PRRX1-TOP2A interaction is a malignancy-promoting factor in human malignant peripheral nerve sheath tumours. Br J Cancer. 2024;130:1493–504. 38448751 10.1038/s41416-024-02632-8 PMC11058259

[54] Nielsen CF Zhang T Barisic M Kalitsis P Hudson DF. Topoisomerase IIα is essential for maintenance of mitotic chromosome structure. Proc Natl Acad Sci U S A. 2020;117:12131–42. 32414923 10.1073/pnas.2001760117 PMC7275761

[55] Dong Y Zuo W Li Y. PKG-mediated phosphorylation of TOP2A activates HDAC to drive photoreceptor cell death in rd1 mouse inherited retinal degeneration. J Neurochem. 2025;169:e70077. 40320856 10.1111/jnc.70077

