# Supplementary material for: Machine learning identified EPHB2 and TOP2A as key genes linking systemic lupus erythematosus to colorectal cancer
Source: Medicine (Baltimore). 2025 Sep 12;104(37):e44521. doi: 10.1097/MD.0000000000044521 (PMC12440411; doi:10.1097/MD.0000000000044521)

**Figure S1.** WGCNA analysis of the GSE41258 cohort. (A) Sample dendrogram and trait heatmap. (B) Scale independence showing the scale-free topology model fit (Model Fitting R²) against different soft threshold (power) values. (C) Mean connectivity of the network across various soft threshold values.


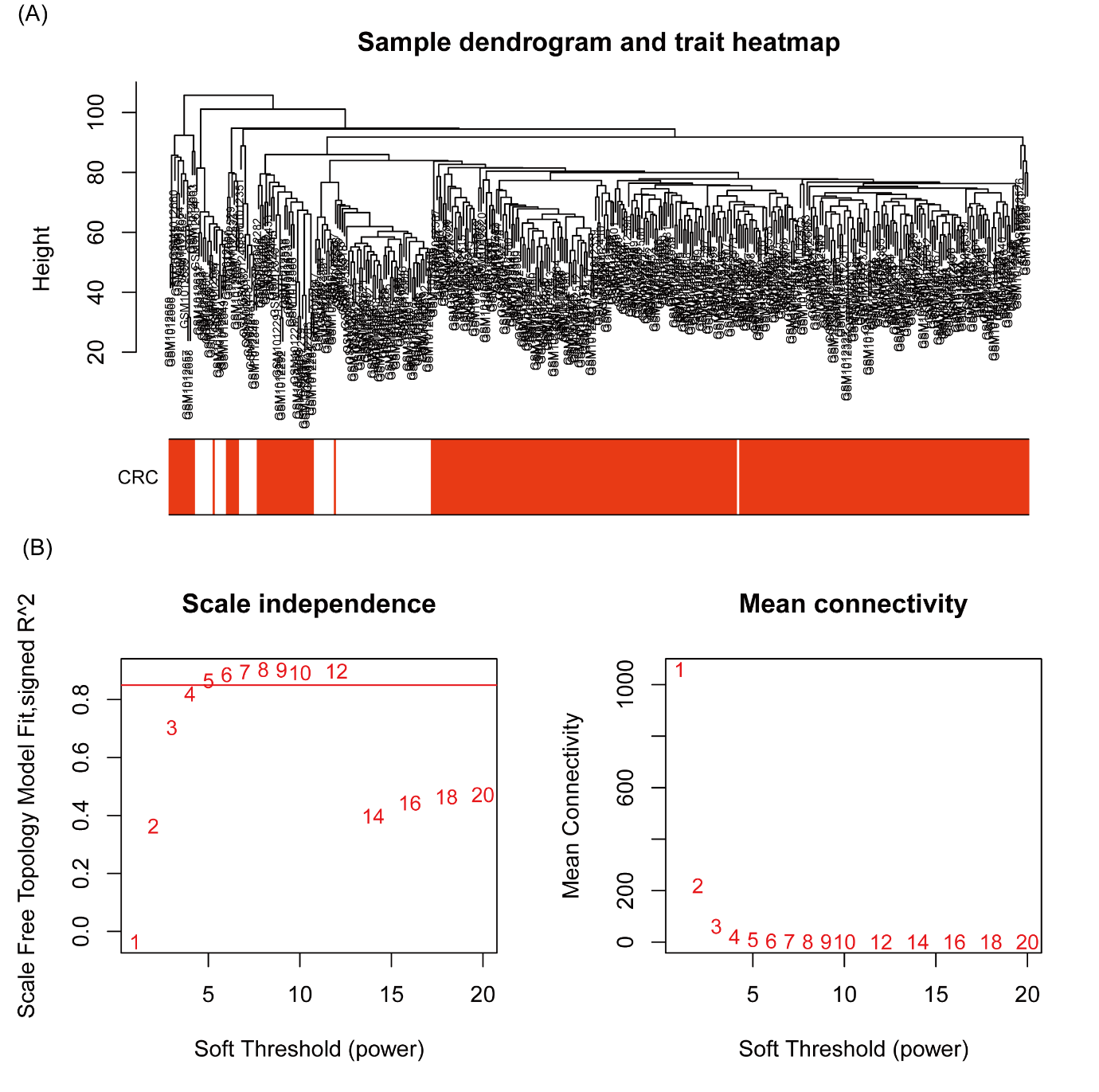


**Figure S2.** WGCNA analysis of the GSE72326 cohort. (A) Sample dendrogram and trait heatmap. (B) Scale independence showing the scale-free topology model fit (Model Fitting R²) against different soft threshold (power) values. (C) Mean connectivity of the network across various soft threshold values.


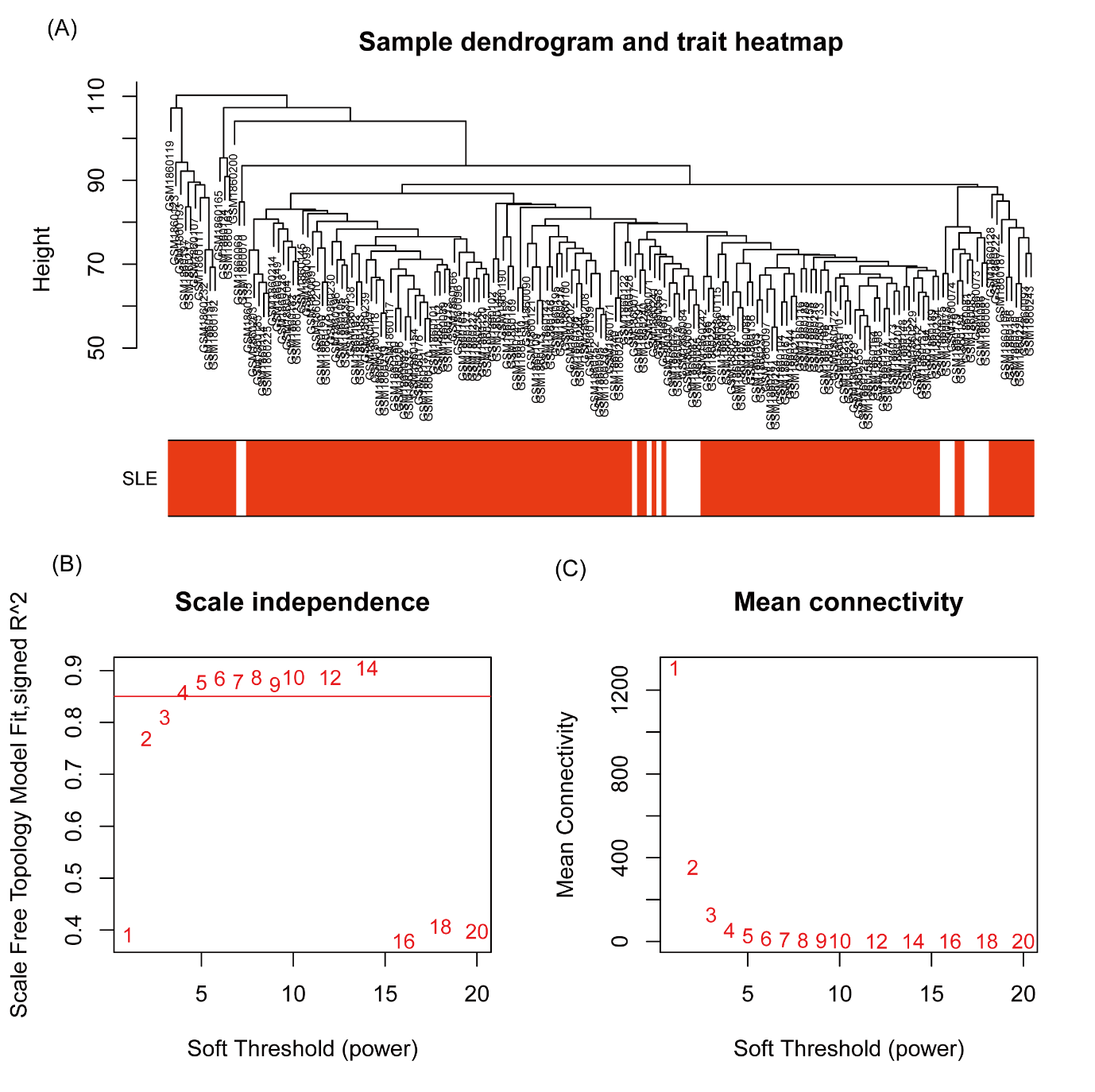

Supplement: Supplementary file 1 [file medi-104-e44521-s001.docx]
